# Dual Processing Model for Medical Decision-Making: An Extension to Diagnostic Testing

**DOI:** 10.1371/journal.pone.0134800

**Published:** 2015-08-05

**Authors:** Athanasios Tsalatsanis, Iztok Hozo, Ambuj Kumar, Benjamin Djulbegovic

**Affiliations:** 1 Comparative Effectiveness Research, University of South Florida, Tampa, FL, United States of America; 2 Department of Internal Medicine, University of South Florida, Tampa, FL, United States of America; 3 Department of Mathematics, Indiana University of Northwest, Gary, IN, United States of America; 4 Departments of Hematology and Health Outcomes and Behavior, H. Lee Moffitt Cancer Center & Research Institute, Tampa, FL, United States of America; University of Louisville, UNITED STATES

## Abstract

Dual Processing Theories (DPT) assume that human cognition is governed by two distinct types of processes typically referred to as type 1 (intuitive) and type 2 (deliberative). Based on DPT we have derived a Dual Processing Model (DPM) to describe and explain therapeutic medical decision-making. The DPM model indicates that doctors decide to treat when treatment benefits outweigh its harms, which occurs when the probability of the disease is greater than the so called “threshold probability” at which treatment benefits are equal to treatment harms. Here we extend our work to include a wider class of decision problems that involve diagnostic testing. We illustrate applicability of the proposed model in a typical clinical scenario considering the management of a patient with prostate cancer. To that end, we calculate and compare two types of decision-thresholds: one that adheres to expected utility theory (EUT) and the second according to DPM. Our results showed that the decisions to administer a diagnostic test could be better explained using the DPM threshold. This is because such decisions depend on objective evidence of test/treatment benefits and harms as well as type 1 cognition of benefits and harms, which are not considered under EUT. Given that type 1 processes are unique to each decision-maker, this means that the DPM threshold will vary among different individuals. We also showed that when type 1 processes exclusively dominate decisions, ordering a diagnostic test does not affect a decision; the decision is based on the assessment of benefits and harms of treatment. These findings could explain variations in the treatment and diagnostic patterns documented in today’s clinical practice.

## Introduction

A paradigmatic decision-making dilemma faced by clinicians is whether to observe the patient without ordering a diagnostic test, order a diagnostic test and act according to the results of the test, or administer treatment without ordering a test. Typically, this decision relies on the probability of disease and the relationship between the treatment’s harms and benefits. As described later in this paper, the assessment of the likelihood of disease and the evaluation of treatment’s benefits and harms is often done intuitively, but this decision-making process can be formalized under the “threshold model”.

**Fig 1:**
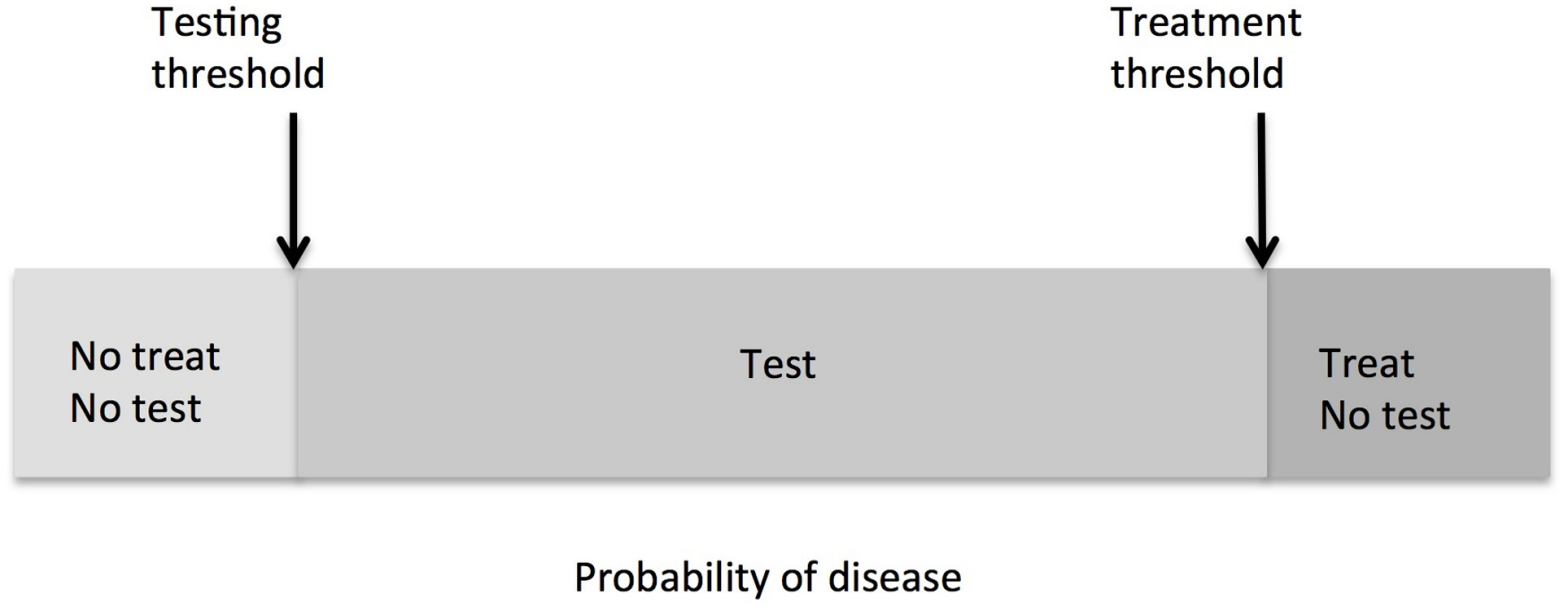
Relation between the probability of disease and the threshold probabilities for testing and treatment (adopted from [3]).

According to the threshold model [1,2], when faced with a choice of observing the patient, ordering a diagnostic test, or administering treatment, there is a probability of disease, also known as threshold probability, at which a decision maker is indifferent between any two choices (e.g. treating vs. ordering a test, or ordering a test vs. withholding treatment) [3–6]. Furthermore, decisions involving diagnostic testing rely on two probabilities of disease known as testing and treatment thresholds. Testing threshold relates to the decision about ordering a test vs. observing a patient and treatment threshold relates to the decision about administering treatment vs. ordering the diagnostic test. According to the threshold model [1,2], if the probability of disease is smaller than the testing threshold, the test should be withheld. If the probability of disease is above the treatment threshold, then treatment should be ordered without ordering a diagnostic test. The test should only be ordered if the estimated probability of the disease is between the testing and treatment thresholds (Fig 1).

The threshold model relies on expected utility theory (EUT) and it was formulated almost 4 decades ago[1,2]. EUT suggests that when choosing between different strategies, the decision maker should always select the strategy that leads to the outcome with the highest expected utility. It has been well documented, however, that EUT is routinely violated by decision-makers [7–9]. These violations are typically attributed to the decision maker’s emotional, experiential or intuitive responses to decision choices that are different from the EUT derived expected utilities. Consequently, the main drawback of threshold model is its reliance on EUT as demonstrated in our recent empirical study [10].

The importance of non-EUT based cognitive processes has recently been highlighted by dual processing theories (DPT) of human reasoning and decision-making [11], which are increasingly accepted as the dominant explanation of how people make decisions [5,7,12–19]. DPT posits that human cognition is governed by two types of processes [11,19]: type 1 processes, which are intuitive, automatic, fast, narrative, experiential and affect-based, and type 2 processes, which are analytical, slow, verbal, deliberative and allow for abstract and hypothetical thinking. Therefore, the EUT model cannot be seen as an adequate model of medical decision-making.

To overcome the drawbacks of the EUT-based threshold model we recently developed a Dual Processing Model (DPM) [12], which is based on DPT. The DPM [12] incorporates regret to model type 1 processes and EUT to model type 2 processes. This is because regret is one of the key emotions that play a major role in medical-decision making [20–22]. Two main assumptions of DPM are that the extent of activation of type 1 processes is regulated by a parameter γ, and that when faced with a decision problem our initial responses tend to rely mostly on type 1 processes [23].

In our previous work [12] we demonstrated the applicability of the DPM-based threshold in a situation when no diagnostic test is available but a clinician has to make a decision whether to administer treatment or not. Here, we extend our work to include a wider class of medical decision-making problems that involve diagnostic testing.

## Methods

### Threshold models

#### EUT threshold model

Most decision theories agree that decision-making depends on evaluation of harms (losses) and benefits (gains) associated with a given decision strategy. The threshold model takes this into consideration by relating the threshold probability to benefit/harms ratio. For example, the EUT threshold is calculated as:

$$p_{t,EUT}=\frac{1}{1+\frac{B_{II}}{H_{II}}},\text{ for }H_{II}>0$$

Where p_t, EUT_ ∈ [0,1] is the threshold probability i.e. the probability of disease at which we are indifferent between treatment vs. no treatment. *B*_*II*_ ≥ 0 is the net benefits of treatment defined as the difference in outcomes of treating and not treating a patient with disease, as realized by type 2 (denoted also as *II* in the equations) processes [12,24–29]. *H*_*II*_ > 0 is the net harms due to treatment, defined as the difference in outcomes of not treating and treating the patients without disease as realized by type 2 processes[12,24–29]. Typically, the values of harms and benefits are obtained from the best available evidence found in the literature [12,24–29]. Note that for the validity of the *p*_*t*,*EUT*_ equation, *H*_*II*_ must take values greater than zero. This requirement is clinically justifiable because in reality every treatment is associated with some harms.

#### Regret-based model

When treating a patient, a decision maker may face two types of regret: regret associated with failure to provide necessary treatment (regret of omission) and regret associated with administering harmful treatment (regret of commission) [12,21,22,30,31]. These two regrets are used to compute the regret based threshold probability as:

$$p_{t,RG}=\frac{1}{1+\frac{B_{I}}{H_{I}}},\text{ for }H_{I}>0$$

where *p*_*t*,*RG*_ ∈ [0,1] is the threshold probability at which a decision maker is indifferent between treating or not a patient. *B*_*I*_ ≥ 0 is the net benefits of treatment as realized by type 1 processes and computed here as regret of omission. *H*_*I*_ > 0 is the net harms of treatment as realized by type 1 (denoted also as *I* in the equations) processes and computed here as regret of commission. [12,21,22]. Both *B*_*I*_ and *H*_*I*_ values may be elicited using the Dual Analogue Scale described elsewhere [21,22]. As with *H*_*II*_, *H*_*I*_ must take values greater than zero so that *p*_*t*,*RG*_ is defined.

#### Dual Processing Model

**Fig 2:**
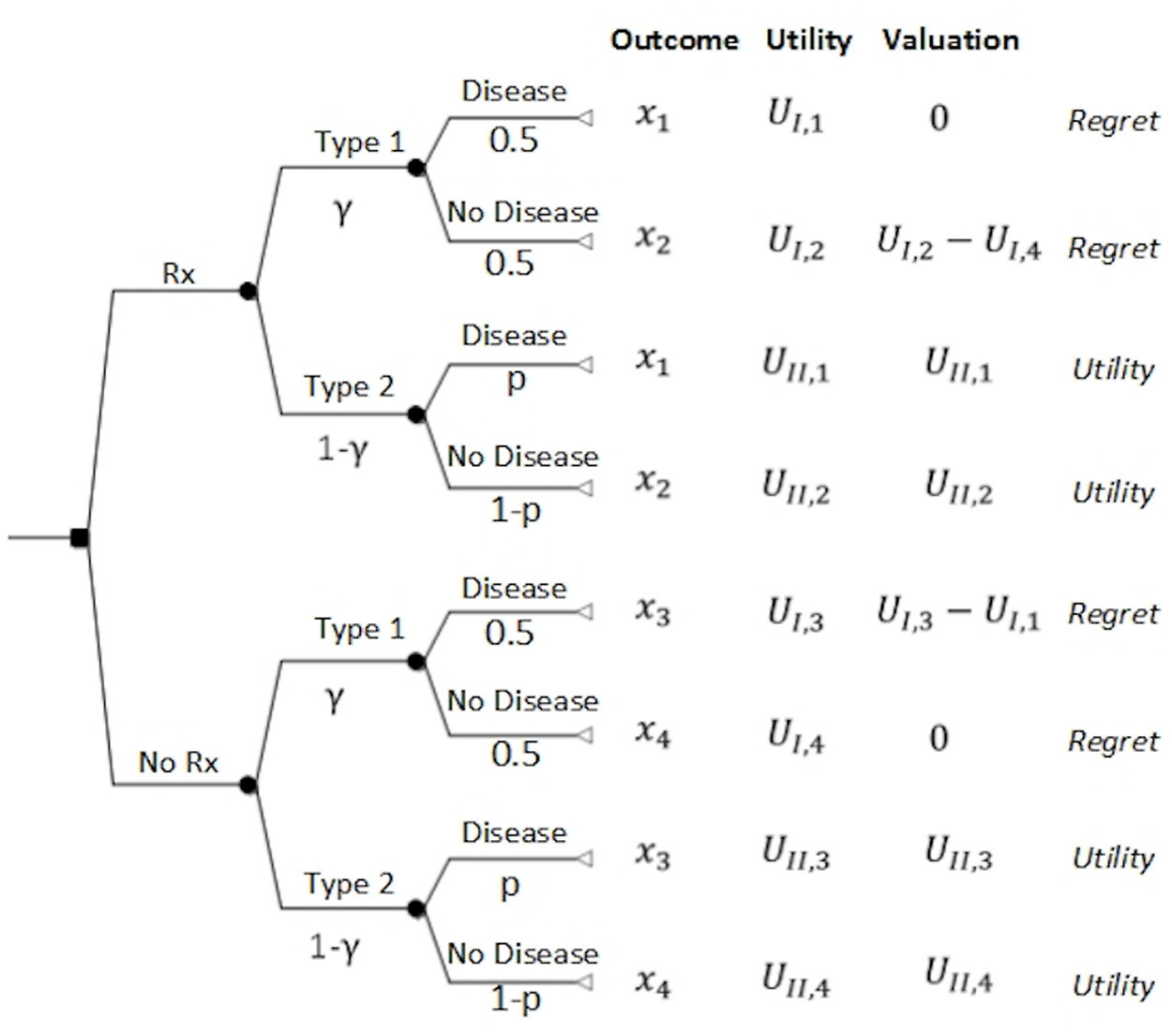
Decision tree describing a typical scenario in which a physician is considering administering (Rx) / withholding treatment (NoRx) to/from his patient. *x*_*i*_ represents an outcome; *γ* is the involvement of type 1 in the decision process; *p* is the probability of disease; *U*_*I*,*i*_ is the utility of the outcome *x*_*i*_ under type 1 process; *U*_*II*,*i*_ is the utility of outcome *x*_*i*_ under type 2 processes; The valuation of an outcome *x*_*i*_ under type 1 is estimated as the regret associated with the outcome *x*_*i*_; the valuation of an outcome *x*_*i*_ under type 2 is estimated as the utility of the outcome *x*_*i*_ ([12] for details).

DPM [12] assumes that the valuation of a risky choice is formed as the combination of type 1 and type 2 processes. To demonstrate, consider a clinical scenario (Fig 2) in which a decision maker is faced with a choice of treating (Rx) or not (NoRx) of a patient who has a disease with probability p. Each decision results in a specific outcome *x*_*i*_. For example, outcome *x*_1_ corresponds to the decision of treating a patient who had a disease and outcome *x*_2_ corresponds to the decision of treating a patient who did not have a disease. The parameters $x_{i}^{m_{I}}\geq 0$ and $x_{i}^{m_{II}}\geq 0$ correspond to valuations of the outcome *x*_*i*_ when the decision maker employs type 1 and 2 processes respectively. Each outcome is also associated with type 1, *U*_*I*,*i*_ ≥ 0, and type 2, *U*_*II*,*i*_ ≥ 0, utilities.

Solving the decision tree in Fig 2, we derive the DPM threshold probability, *p*_*t*_ ∈ [0,1], or the probability at which we are indifferent between providing and withholding treatment, as [12]:

$$p_{t}=\text{min}\left\{\left(p_{t,EUT}\right)\left[1+\frac{\gamma }{2\left(1-\gamma \right)}\left(\frac{H_{I}}{H_{II}}\right)\left(1-\frac{B_{I}}{H_{I}}\right)\right],1\right\},\;\;for\;\gamma \in \left[0,\;1\right]$$ (1)

The interaction between type 1 and type 2 processes is represented by the parameter *γ* ∈ [0,1]. *γ* exemplifies the extent of activation of type 1 processes in the decision in such a way that when it is zero the decision-making processes is based on type 2 processes according to the EUT paradigm. As the value of *γ* increases, so does the involvement of type 1 processes in the decision. However, Eq 1 is not valid for values of *γ* = 1. In that case, based on the decision tree depicted in Fig 2 for *γ* = 1, the decision to treat or not depends solely on the explicit evaluation of harms and benefits based on type 1 processes (see also the Special Case in S1 Appendix). As a consequence, treatment should be administered only if benefits of treatment as assessed by type 1 processes outweigh harms of treatment.

*γ* can be best visualized as the relative distance between the analytically derived threshold *p*_*t*,*EUT*_ and the regret derived threshold *p*_*t*,*RG*_, or [32]:

$$\gamma =min\left\{\frac{\left|p_{t,EUT}-p_{t,RG}\right|}{p_{t,EUT}},1\right\}$$

However, *γ* can be affected by many different mechanisms that characterize type 1 processes. Even though our model assumes a dominant role of regret, it does also incorporate other mechanisms of type 1 cognitive processes.

If a patient’s probability of disease is greater than the threshold probability then the decision maker favors treatment and withholds treatment otherwise. Eq 1 shows the impact of the extent of treatment harms and benefits on decisions and how they relate to the DPM and EUT thresholds. When the type 1 benefits of treatment as perceived by the decision maker are higher than its harms, the DPM threshold is **always lower** than the EUT threshold. Conversely, the DPM threshold is **always greater** than the EUT threshold if the type 1 harms of treatment are perceived to be higher than benefits. These changes in the threshold often lead to different choices than those predicted by the EUT and therefore may explain the violations of EUT in decision-making described extensively in literature [7,8,21,22,33,34].

#### DPM with a diagnostic test

In many cases the use of a diagnostic test may assist the treating physician in decreasing diagnostic uncertainty. However, obtaining diagnostic information may expose the patient to unnecessary risks [4] and therefore, a test should be ordered only when benefits of testing outweigh its risks [3].

Typically, deciding when to perform a diagnostic test relates to the assessment of the prior probability that a patient has a suspected disease [3]. If the probability of disease is very low or very high, then performing a diagnostic test may be unnecessary. As explained above, according to the threshold framework, there exists: 1. a probability at which we are indifferent between performing a diagnostic test and withholding treatment; and 2. a probability at which we are indifferent between performing a diagnostic test and administering treatment. These probabilities are formally known as the *threshold probabilities for testing* and they are decomposed into 1. *testing threshold* (*p*_*tt*_) and 2. *treatment threshold* respectively (*p*_*rx*_) [3]. Here we derive and present both threshold probabilities in terms of DPM [12].

**Fig 3:**
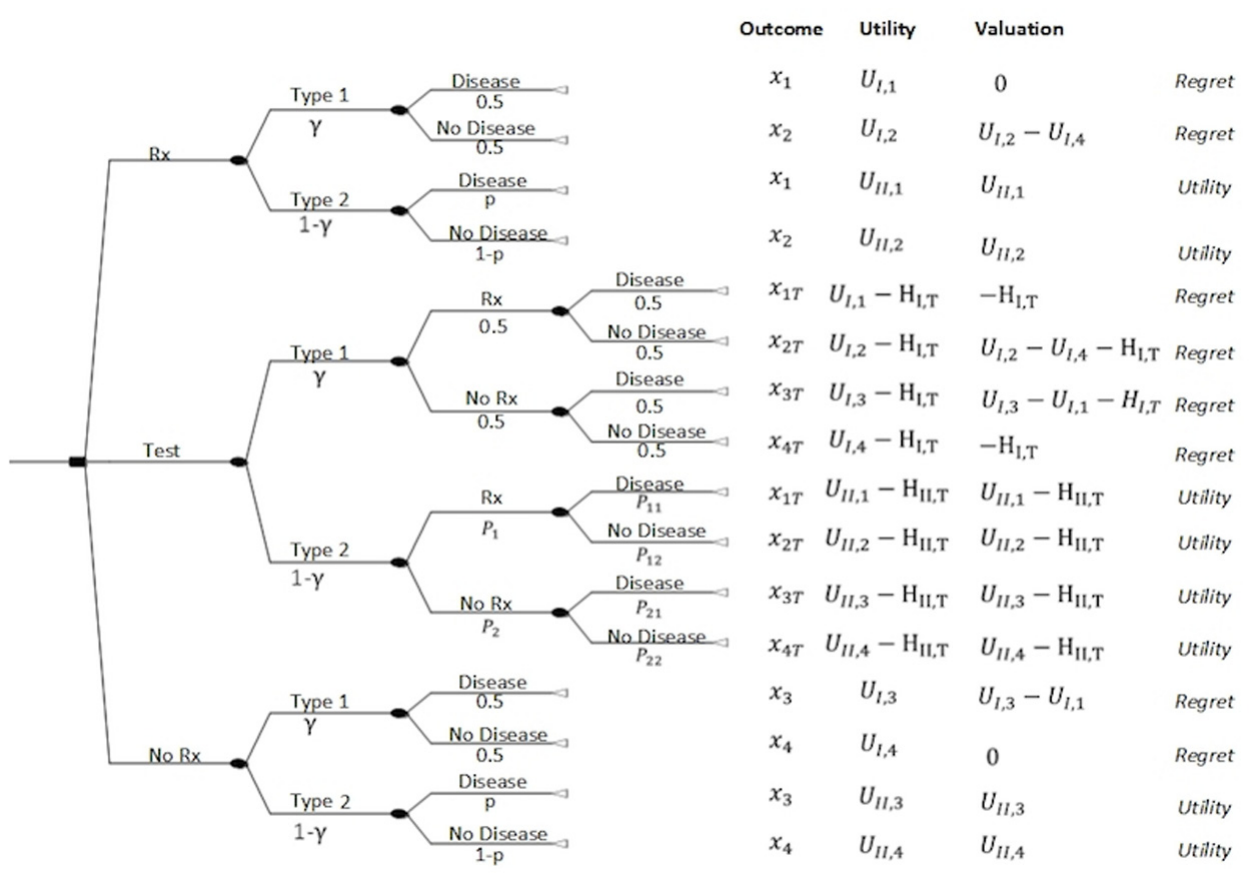
Decision tree describing a typical scenario in which a physician is considering one the following three strategies: administering treatment (Rx); withholding treatment (NoRx); and performing a diagnostic test before deciding on treatment (Test). *x*_*i*_ represents an outcome; *γ* is the involvement of type 1 in the decision process; *p* is the probability of disease; *U*_*I*,*i*_ is the utility of the outcome *x*_*i*_ under type 1 and *U*_*II*,*i*_ is the utility of outcome *x*_*i*_ under type 2 cognitive processes; *H*_*I*,*T*_ denotes the harms of test as realized by type 1 and *H*_*II*,*T*_ denotes the harms of test as realized by type 2 processes; *P*_1_ = *pS*+(1-*p*)(1-*S*_*p*_); *P*_11_ = *pS*/*P*_1_; *P*_12_ = (1-*p*)(1-*S*_*p*_)/*P*_*1*_; *P*_*2*_ = (1-*p*)*S*_*p*_+*p*(1-*s*); *P*_21_ = *p*(1-*s*)/*P*_2_; *P*_22_ = (1-*p*)*S*_*p*_/*P*_2_; *S* is the test’s sensitivity and *S*_*p*_ the test’s specificity. The valuation of an outcome *x*_*i*_ under type 1 is estimated as the regret associated with the outcome *x*_*i*_; the valuation of an outcome *x*_*i*_ under type 2 is estimated as the utility of the outcome *x*_*i*_.

We consider a generic scenario in clinical decision-making in which a decision maker is considering one of three strategies for the management of a patient’s condition (Fig 3). These strategies are: 1. do nothing (NoRx), 2. perform a diagnostic test (T), and 3. administer treatment (Rx). The patient may have a disease (D) with probability *p*. Each strategy results in an outcome *x*_*i*_, which is associated with a certain valuation, $x_{i}^{m_{I}}\geq 0$ when type 1 processes are involved and $x_{i}^{m_{II}}\geq 0$ when type 2 processes are employed. Each outcome has a utility *U*_*I*,*i*_ ≥ 0 for type 1 processes and *U*_*II*,*i*_ ≥ 0 for type 2 processes. As described earlier, valuation of outcomes under type 1 processes is performed using regret elicited using the Dual Visual Analogue Scale (DVAS) while valuation of outcomes under type 2 processes is based on EUT and the latest available evidence [12].

Solving the decision tree in Fig 3, we derive the following expected valuations for each strategy (detailed derivation is presented in the S1 Appendix):

$$V\left(Rx\right)=\frac{\gamma }{2}\left(U_{I,2}-U_{I,4}\right)+\left(1-\gamma \right)\left[pU_{II,1}+\left(1-p\right)U_{II,2}\right]$$

$$V\left(NoRx\right)=\frac{\gamma }{2}\left(U_{I,3}-U_{I,1}\right)+\left(1-\gamma \right)\left[pU_{II,3}+\left(1-p\right)U_{II,4}\right]$$

and

$$V\left(T\right)=\frac{\gamma }{4}\left(U_{I,2}-U_{I,4}+U_{I,3}-U_{I,1}\right)+\left(1-\gamma \right)\left[pSU_{II,1}+\left(1-p\right)\left(1-S_{p}\right)U_{II,2}+p\left(1-S\right)U_{II,3}+\left(1-p\right)S_{p}U_{II,4}\right]-\left(\gamma H_{I,T}+\left(1-\gamma \right)H_{II,T}\right)$$

The notation for the expected valuations is as follows: *U*_*I*,*i*_ ≥ 0 and *U*_*II*,*i*_ ≥ 0 corresponds to the utilities of the *x*_*i*_ outcome under type 1 and 2 processes respectively; *p* is the probability of disease; *γ* ∈ [0,1] is the weight given to type 1 processes; *S* ∈ [0,1] is the sensitivity of the diagnostic test; *S*_*p*_ ∈ [0,1] is the specificity of the diagnostic test; *H*_*I*,*T*_ ≥ 0 and *H*_*II*,*T*_ ≥ 0 denote the harms associated with the diagnostic test as perceived by type 1 and 2 processes respectively.

### Threshold probabilities for testing

#### Testing threshold

The testing threshold is the probability at which we are indifferent between withholding treatment and ordering a diagnostic test [3]. Thus, working with the expected valuations for NoRx and T, *V*(*NoRx*) = *V*(*T*), and solving for the threshold probability we derive:

$$p_{tt}=min\left\{p_{tt,EUT}\left[1+\frac{\gamma }{4\left(1-\gamma \right)\left(1-S_{p}\right)\left(1+\frac{1}{1-S_{p}}\frac{H_{II,T}}{H_{II}}\right)}\left(\frac{H_{I}}{H_{II}}\left(1-\frac{B_{I}}{H_{I}}\right)+4\frac{H_{I,T}}{H_{II}}\right)\right],1\right\},\;\;\;for\;\gamma \in \left[0,1\right]$$ (2)

Eq 2 is invalid for *γ* = 1. In that case, decision makers should always choose not to treat instead of testing i.e. ordering a diagnostic test does not contribute to the decision (see S1 Appendix Special Case section for details). This result is a function of type 1 processes, which do not have a role in calibration of probabilities, but treat each choice as “yes/no” outcome (see Fig 3). Indeed, the key role of a diagnostic test is to decrease uncertainty by increasing/decreasing probability of disease. When a decision-maker does not take this probability into account, then there is no sense in considering a diagnostic test.

If the probability of disease is greater than or equal to *p*_*tt*_ the decision maker favors the diagnostic test; otherwise, he/she prefers withholding treatment. The DPM testing threshold (Eq 2) is **always higher** than the analytically derived EUT testing threshold if the relationship between type 1 benefits and harms of treatment and harms of test is *H*_*I*_+4*H*_*I*,*T*_ > *B*_*I*_. This relationship shows that if the harms of test and treatment are perceived greater than the benefits of treatment, the decision maker requires more certainty before testing. Conversely, the DPM testing threshold is **always lower** than the EUT testing threshold if *H*_*I*_+4*H*_*I*,*T*_ > *B*_*I*_, which demonstrates that the decision maker requires less certainty before testing. Note that the accuracy of the diagnostic test (expressed in terms of sensitivity and specificity) does not affect this finding. Both *p*_*tt*,*EUT*_ and *p*_*tt*_ are undefined for the special case of *S*_*p*_ = 100%. However, as *S*_*p*_→100%, *p*_*tt*,*EUT*_→1 and *p*_*tt*_→1. This finding demonstrates a decision maker’s aversion in providing diagnostic testing, which is perceived to lead to more harms than benefits. Note that the values of *γ*, *S*_*p*_, *S* and $\left(\frac{H_{I}}{H_{II}}\right)$ affect the degree (“depth”) by which the DPM testing threshold *p*_*tt*_ is greater/lower than the classic EUT threshold *p*_*tt*,*EUT*_; however, they do not change the quality of the relationship.

#### Treatment threshold

The treatment threshold is the probability at which we are indifferent between testing and administering treatment [3]. Working with the expected valuations of Rx and T, *V*(*Rx*) = *V*(*T*), and solving for the threshold probability we derive:

$$p_{rx}=min\left\{p_{rx,EUT}\left[1+\frac{\gamma }{4\left(1-\gamma \right)S_{p}\left(1-\frac{1}{S_{p}}\frac{H_{II,T}}{H_{II}}\right)}\left(\frac{H_{I}}{H_{II}}\left(1-\frac{B_{I}}{H_{I}}\right)-4\frac{H_{I,T}}{H_{II}}\right)\right],1\right\},\;\;for\;\gamma \in \left[0,1\right]$$ (3)

Eq 3 is invalid for *γ* = 1. In that case, decision makers should always choose treating instead of testing (see S1 Appendix Special Case section for details). As outlined above, this result is a consequence of how type 1 processes work: by treating each choice as “yes/no” outcome there is no sense in taking diagnostic test probabilities into account (see Fig 3).

If the probability of disease is greater than or equal to *p*_*rx*_ the decision maker will choose to administer treatment; otherwise, he/she will prefer to perform a diagnostic test. The DPM treatment threshold (Eq 3) is **always higher** than the analytically derived EUT treatment threshold if the relationship between type 1 benefits and harms of treatment and harms of test is as follows: *H*_*I*_ > *B*_*I*_+4*H*_*I*,*T*_. This relationship shows that if the decision maker assumes that the harms of treatment are higher than its benefits added to the harms of testing, then he requires more certainty before proceeding with treatment. Conversely, the DPM treatment threshold is **always lower** than the EUT treatment threshold if *H*_*I*_ < *B*_*I*_+4*H*_*I*,*T*_, which demonstrates that the decision maker requires less certainty before proceeding with treatment. As above, the test sensitivity and specificity does not affect this relationship. Both rules assume that the diagnostic test is objectively assessed (via type 2 functioning) to be less harmful than the treatment, *H*_*II*,*T*_ < *H*_*II*_, which is almost always the case. The values of *γ*, *S*_*p*_, *S*, and $\left(\frac{H_{I}}{H_{II}}\right)$ affect the extent (“depth”) by which the dual threshold *p*_*rx*_ is greater/lower than the classic EUT threshold *p*_*rx*,*EUT*_ but does not change the quality of the relationship.

To summarize, for *γ* ∈ [0,1) a decision maker will choose to perform a diagnostic test if the patient’s probability of disease is *p*_*tt*_ ≤ *p* < *p*_*rx*_. The probabilities *p*_*tt*_ and *p*_*rx*_ are functions of a decision maker’s attitudes towards treatment benefits and harms as well as harms of testing and they are derived using both type 1 and type 2 cognitive mechanisms. The probability of disease, *p* can be estimated by statistical evidence, and by the physician’s intuition and experience. When *γ* = 1, the management choices are limited to treatment vs no treatment as testing results in the overall lower valuation in comparison with the other two alternatives. Therefore, the optimal decision is a function of the decision maker’s attitudes towards treatment benefits and harms as assessed by type 1 processes (see S1 Appendix Special Case section for details).

## Case Study

We will now demonstrate the applicability of the proposed method as it relates to decisions regarding performing prostate biopsy in a patient suspected of having prostate cancer. Concerns about prostate cancer may be raised by elevated values of the Prostate-specific Antigen (PSA) biomarker and/or by abnormalities found through Digital Rectal Examination (DRE). Further verification is obtained through a biopsy, which is currently the gold standard for diagnosis of prostate cancer. During the prostate biopsy, several needles are inserted through the rectum wall into the areas of the prostate, where the abnormality is detected, to remove small amounts of tissue, which are later analyzed in the lab. The patient may experience discomfort, pain, bleeding, hematuria, infections, sepsis and vasovagal episodes as a result of the biopsy[35–40]. Table 1 summarizes the risks and benefits associated with prostate biopsy as reported in literature.

**Table 1:** Harms associated with cancer biopsy as reported in literature*.

Cancer biopsy	
Harm (H)	Size
**Death (H)**	0.09% [52]
**Infections (H)**	2–3% [37–39]
**Hematuria (H)**	50%-60% [35,36,38]
**Discomfort, pain, bleeding, sepsis, vasovagal episodes (H)**	<10% [35–40]

* The data are related to transrectal ultrasound guided prostate biopsy.

Contingent on the results of the biopsy, the treating urologist may choose to perform a radical prostatectomy and surgically remove a part or all of the prostate gland. The goal of the procedure is to cure or control the cancer. The procedure is performed either through an open surgery, where the surgeon makes a cut in the abdomen or between the testicles and the back passage, or laparoscopy, where the surgeon makes several small incisions in the pelvis. In both cases, the patient may experience major or minor complications after or during the surgery including heart problems, blood clots, blood loss, allergic reactions to anesthesia, infections, erectile dysfunction, urinary incontinence, damage to the urethra or the rectum [41–43]. However, radical prostatectomy has statistically beneficial effect on patient’s survival compared to observation[44–46]. Table 2 summarizes the risks and benefits of radical prostatectomy as reported in literature.

**Table 2:** Benefits and harms of radical prostatectomy as reported in literature*.

Radical prostatectomy	
Benefit (B) or Harm (H)	Size
**Survival (B)**	Absolute risk reduction: 2.5%- 10% [44–46]
**Death (H)**	0.4% [46]
**Erectile dysfunction (H)**	37% [46]
**Urinary incontinence (H)**	10.8% [46]
**Heart problems (H), blood clots (H), blood loss (H), allergic reactions to anesthesia (H), infections (H), damage to the urethra or the rectum (H)**	<10% [41–43]

* The data are related to radical prostatectomy performed as an open surgery or laparoscopic.

For example consider the management strategies for a 66-year-old patient with elevated PSA and abnormal DRE: 1. do nothing (e.g. observe or wait for 6 months to repeat PSA and DRE), 2. perform a prostate biopsy and act accordingly, and 3. proceed directly with radical prostatectomy. Based on the evidence provided on Tables 1 and 2 we define the type 2 benefits and harms regarding radical prostatectomy as *B*_*II*_ = 2.5% to 10% and *H*_*II*_ = 0.4%. The harms associated with prostate biopsy are *H*_*II*,*T*_ = 0.09%.

For demonstration and simplification purposes, we will first describe how the decision dilemma described above would be solved relying solely on type 2 processes focusing on the most important harms and benefits i.e. those related to survival. An elaborate modification of the EUT model to include all harms and benefits reported in literature can also be implemented as in [24]. We assume that the values of sensitivity and specificity of the biopsy are equal to 86% and 94% respectively [47] (the highest reported values for biopsy guided by transrectal ultrasound (TRUS)). The EUT-based threshold probabilities, are derived by Eqs 2 and 3 (assuming *γ* = 0) and they are equal to *p*_*tt_EUT*_ = 0% and *p*_*rx_EUT*_ = 21% (considering maximum benefit of treatment *B*_*II*_ = 10%). The results show that a decision maker will accept biopsy and surgery at very low probabilities of prostate cancer: 0% for biopsy and 21% for surgery. That is, according to the EUT model we should perform biopsy at the slightest suspicion of prostate cancer (i.e., as long as it is greater than 0%!), and can recommend surgery at the estimated probability of prostate cancer > 21%! No physician (or, a patient) would agree with such recommendations. The finding based on the EUT model also contradicts the influential, National Cancer Network (NCCN) expert guidelines for prostate cancer [48] which indicates that a prostate biopsy should be performed if the probability of prostate cancer exceeds 48% (indirectly computed by a Gleason score of 8 or higher for symptomatic patients which translates into 48% according to [49]).

Our finding of low threshold for the action may be attributed to the oversimplified assumption focusing only on mortality, which is rather small: harms (death) attributed to biopsy (0.09%) and prostatectomy (0.4%). If instead of death due to prostatectomy, we focus on erectile dysfunction (37%), which reflects the main concern of patients with 20 or more years of life expectancy, the threshold values increase considerably: *p*_*tt_EUT*_ = 21% and *p*_*rx_EUT*_ = 49% (considering maximum benefit of treatment *B*_*II*_ = 10%). In this case a decision maker opts out of biopsy for probabilities less than 21% and requires more certainty for prostatectomy (49%). The problem, however, is how to integrate multiple outcomes in the EUT model, particularly since it is believed that the values people attach to different outcomes depend on the type 1 mechanisms, which processes the information on all benefits and harms in holistic fashion [4].

It is, therefore, necessary to arrive at decisions using cognitive mechanisms that employ both type 2 and type 1 processes. In our model, this is easily accomplished by increasing the value of *γ*, which reflects the extent of type 1 processes in the decision process. As a result both testing thresholds change. To compute the threshold values from Eqs 2 and 3 we need to elicit the decision maker’s preferences towards biopsy (*H*_*I*,*T*_) and towards prostatectomy (*B*_*I*_,*H*_*I*_). In contrast to the valuation of outcomes through EUT that entail inquiries for every harm and benefit individually, the type 1 processes valuate outcomes in a holistic manner by eliciting regrets of omission and commission using the DVAs [21,22]. Because type 1 processes are unique to each decision-maker, we expect that the DPM-based thresholds will vary among different individuals.

**Fig 4:**
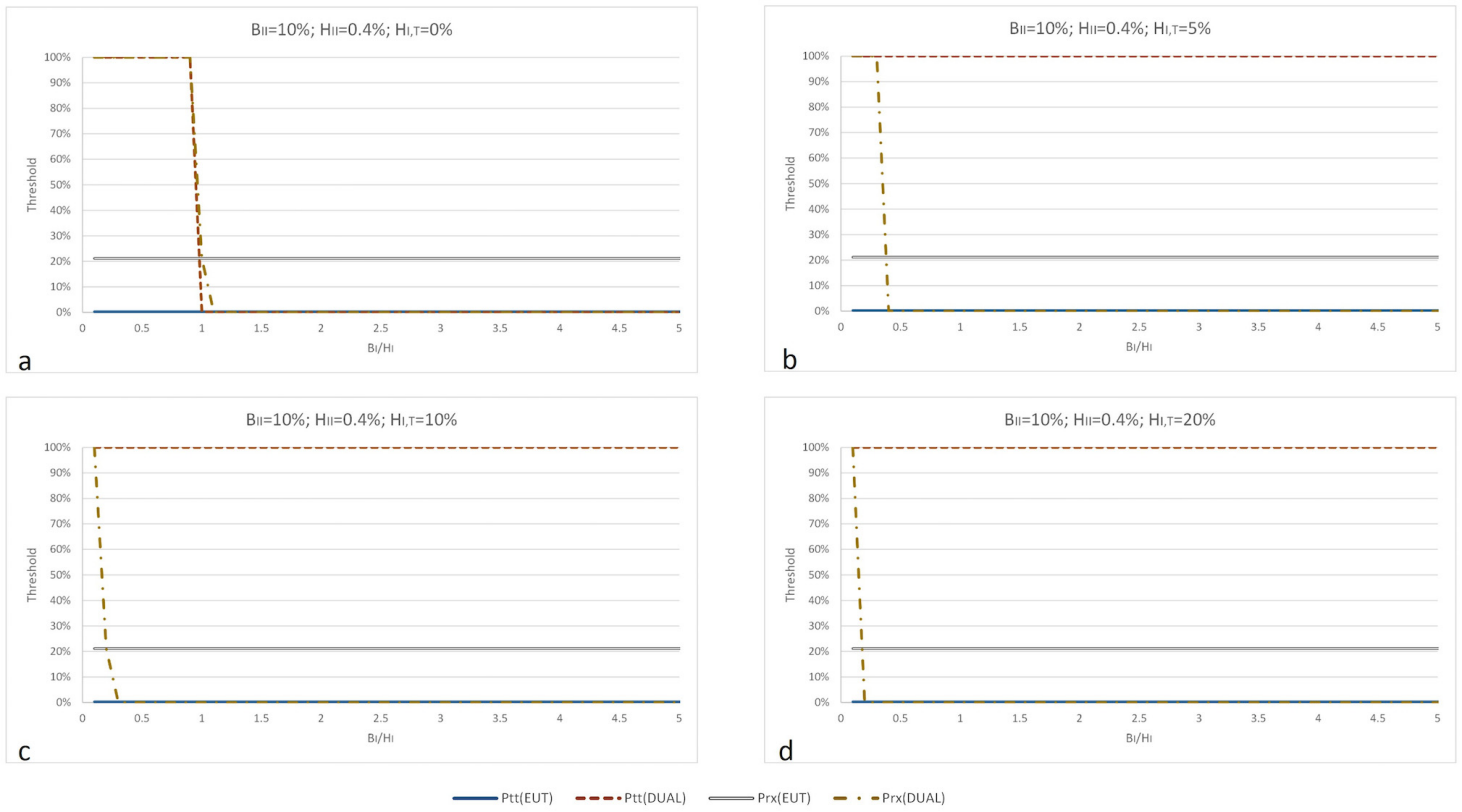
EUT and DPM testing thresholds as functions of type 1 benefits/harms of prostatectomy ratio. The chart progression (Fig 4a–4d) shows the effect of increasing type 1 harms of biopsy on the values of testing thresholds. Unlike the EUT threshold, as harms of biopsy (*H*_*I*,*T*_) increase (Fig 4b, 4c and 4d), the DPM testing threshold increases to the maximum indicating that a decision maker will never choose a biopsy. When benefits of prostatectomy are higher than its harms (*B*_*I*_>*H*_*I*_), the decision maker opts for prostatectomy at practically 0% of disease. Note that the DPM model allows for the treatment threshold to be lower than the testing threshold. This is rationally not possible within the EUT framework, but has been observed in clinical practice. As an illustration consider the case where *B*_*I*_<*H*_*I*_. The DPM testing threshold (*p*_*tt*_) is always higher than the EUT testing threshold (*p*_*tt*,*EUT*_). This is because the DPM testing threshold considers the decision maker’s attitudes towards treatment according to which the benefits of treatment are higher than its harms (e.g. *B*_*II*_>*H*_*II*_). The same holds for the case of *B*_*II*_<*H*_*II*_, but only when *H*_*I*,*T*_>0 (i.e. the diagnostic test is harmful) (Fig 4b, 4c and 4d). If *H*_*I*,*T*_ = 0 and *B*_*II*_>*H*_*II*_ (Fig 4a), the decision maker may choose test or treatment at the same probability of disease. Also, for most *B*_*I*_/*H*_*I*_, the DPM treatment threshold (*p*_*rx*_) is lower than the EUT treatment threshold (*p*_*rx*,*EUT*_). Again, this is because the decision maker values treatment benefits higher that its harms.

**Fig 5:**
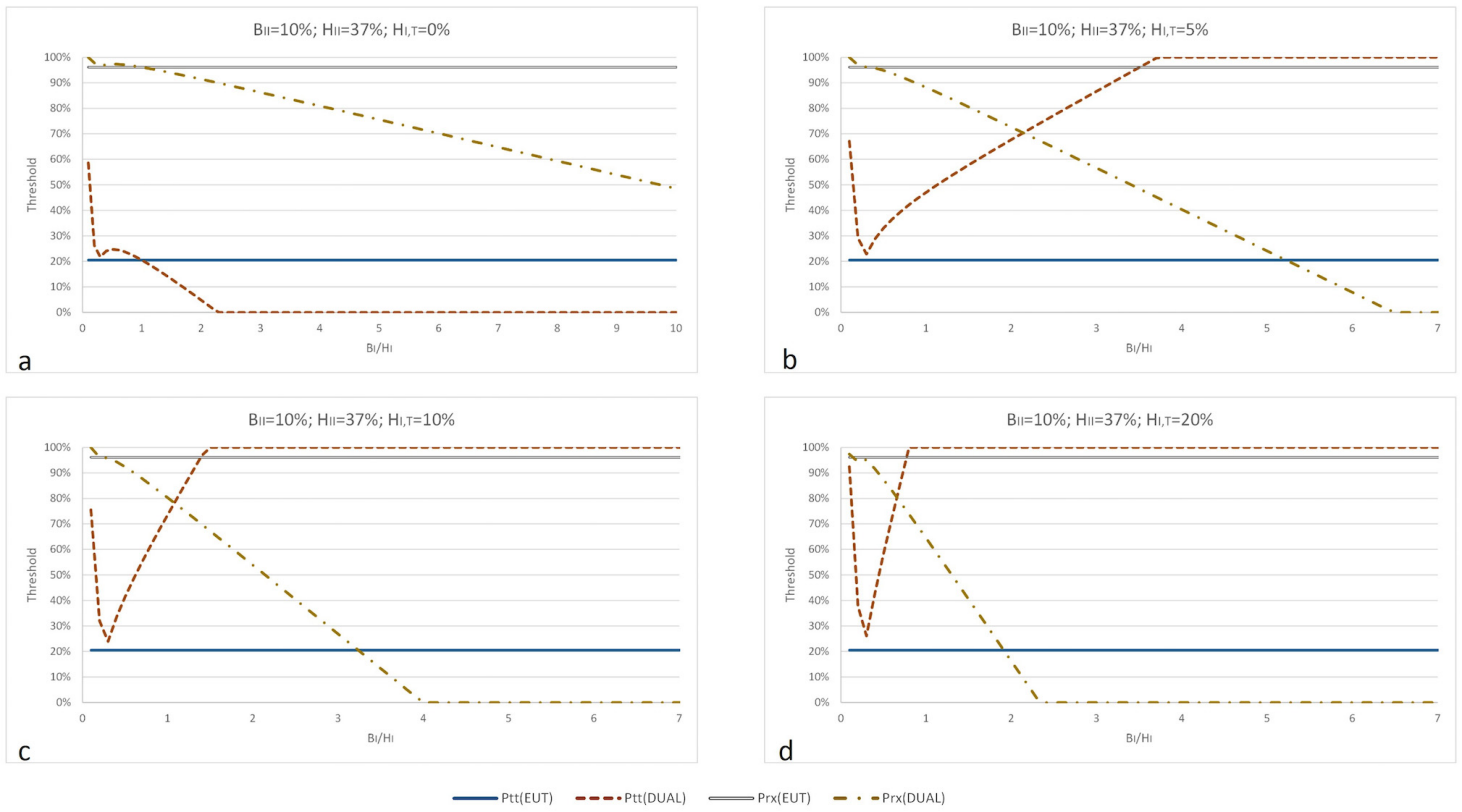
EUT and DPM testing thresholds as functions of type 1 benefits/harms of prostatectomy ratio. The chart progression (Fig 5a–5d) shows the effect of increasing type 1 harms of biopsy to the values of testing thresholds. The value of treatment threshold decreases as the ratio benefit/harms of prostatectomy increases (Fig 5a, 5b, 5c and 5d). The value of testing threshold also decreases as the ratio benefit/harms of prostatectomy increases but only when the harms of biopsy are zero (Fig 5a). If the decision maker perceives biopsy as harmful (Fig 5b, 5c and 5d) the testing threshold increases to the point that he will never choose biopsy. A prostatectomy becomes the preferred choice when *B*_*I*_ > 2*H*_*I*_ in Fig 5b; *B*_*I*_ > *H*_*I*_ in Fig 5c; *B*_*I*_ > 0.8*H*_*I*_ in Fig 5d.

Figs 4 and 5 graph the values of the EUT and DPM thresholds for testing as functions of the type 1 treatment benefit/harm ratio (*B*_*I*_/*H*_*I*_) for different values of type 1 harms of biopsy (*H*_*I*,*T*_). Both figures are generated for maximum benefit of treatment (*B*_*II*_ = 10%) however, Fig 4 assumes harms of treatment relate to survival (*H*_*II*_ = 0.4%) and Fig 5 assumes harms of treatment relate to erectile dysfunction (*H*_*II*_ = 37%).

Fig 4 demonstrates that as the harms of biopsy (*H*_*I*,*T*_) increase (Fig 4b, 4c and 4d), the decision maker will always choose a minimal risk and high benefit prostatectomy over the biopsy. This result is true for any type 1 treatment benefit/harm ratio (*B*_*I*_/*H*_*I*_). In addition, it is shown that the treatment threshold decreases dramatically as the type 1 benefits of prostatectomy outweigh its harms (*B*_*I*_ > *H*_*I*_) and that there exists a benefits/harms ratio at which a decision maker would opt for prostatectomy at practically 0% probability of prostate cancer. These results appear to be different than those based on the EUT model, which recommends biopsy to practically all patients and prostatectomy to the patients with probability of cancer greater than 21% regardless of the decision maker’s preferences towards biopsy and prostatectomy. Note the important result in Fig 4: The treatment threshold is lower than the testing threshold, which is rationally not possible within the EUT framework, but has been observed in clinical practice [20].

The results shown in Fig 5 are far more complicated and fascinating since in this case we consider that the prostatectomy may cause erectile dysfunction in 37 out of 100 people (*B*_*II*_ < *H*_*II*_). It would be logical to assume that a decision maker would always prefer to undergo a diagnostic test with minimal harms before a harmful intervention. This behavior is demonstrated in Fig 5a, where it is assumed that *H*_*I*,*T*_ = 0. However, the prostate cancer probability levels at which a decision maker would opt in for either strategy changes based on the way the decision maker experiences type 1 benefits and harms of prostatectomy. For example, in Fig 5a, the decision maker will accept biopsy at a probability of prostate cancer greater than 40% (closer to NCCN guidelines than the EUT threshold) and prostatectomy for probability greater than 90% if he perceives type 1 harms of prostatectomy as greater than its benefits (e.g. *H*_*I*_ = 8*B*_*I*_). If the decision maker perceives type 1 benefits of prostatectomy higher than its harms (e.g. *B*_*I*_ = 8*H*_*I*_), he may tolerate biopsy for any probability of prostate cancer and accepts prostatectomy at a probability greater than 60%.

Furthermore, as the harms of biopsy (*H*_*I*,*T*_) increase (Fig 5b, 5c and 5d), there is a range of benefit/harms ratio above which the decision maker will prefer prostatectomy over biopsy. For example, when type 1 harms of biopsy are believed to be 10% (Fig 5b), a prostatectomy is preferred to a biopsy (if type 1 benefits of prostatectomy are at least 2 times higher that its harms). Similarly, when *H*_*I*,*T*_ = 20% (Fig 5c), a prostatectomy is preferred to a biopsy if its benefits are slightly higher than its harms. Once again, this is rationally not possible within the EUT framework[20], however, it is observed in current urological practice.

Our case study clearly illustrates that the estimation of the exact values for each parameter, particularly those valuated under type 1 functioning, is not a simple exercise and reflect the complexity of real time decision making. It also demonstrates a variation in decisions made by different decision makers, a finding that may explain why people violate EUT.

## Discussion

In this article we described the derivation of a DPM for medical decision making to accommodate decisions that involve diagnostic testing. Our model is based on Dual Processing Theories and assumes that human cognition relies on type 1 processes, which are intuitive and affect-based, as well as type 2 processes, which are analytical and deliberate processes.

Most medical decision making models rely on EUT and have failed to explain variations between the predicted versus the actual decisions people make. We hypothesize that this is because most existing models depend only on the analytical process of human cognition and ignore other experiential aspects of the decisions humans face[12,50].

Our DPM modifies the EUT threshold model for decision making to incorporate influences by both modes of human cognition: intuitive, affect-based (type 1) and analytical processes (type 2). The derived expressions (Eqs 2 and 3) show that the DPM thresholds modify the EUT thresholds based on the way decision makers evaluate trade-offs of treatment. For example, the DPM-based testing threshold is always higher than the EUT-based threshold when harms of treatment and of test are assessed by type 1 processes to be higher than benefits of treatment. On the other hand, the DPM-based treatment threshold is always lower than the EUT-based threshold when harms of treatment are perceived by the type 1 processes to be lower than treatment benefits and biopsy harms. As described in the Methods section, the test sensitivity and specificity does not affect this relationship. The importance of our findings is best seen in the context of the current attempts to curb waste associated with over-testing. The American Board of Internal Medicine’s (ABIM) nine specialty societies representing 374,000 physicians developed a list of each specialty’s ‘Top Five’ inappropriately prescribed diagnostic tests in order to improve care by eliminating unnecessary tests and procedures [51]. For example, one typical recommendation reads, “Don’t order annual electrocardiograms (EKGs) or any other cardiac screening for low-risk patients without symptoms. False-positive tests are likely to lead to harm through unnecessary invasive procedures, over-treatment and misdiagnosis.” The problem with this guideline is how to determine how low is “low” (in terms of “low-risk”) and how likely is “likely” (in terms of false positives)? That is, at which threshold probability the test should actually be ordered? As illustrated in this paper, because the test characteristics do not affect the thresholds, we do not need to worry about false-positives (or, false-negatives for that matter). What matters is 1) objective data on treatment benefits and harms, and 2) how the decision-maker perceives these data (via type 1 processes). Therefore, a solution to the current health care waste could be to continue emphasizing the need for reliable, evidence-based resources and to highlight the importance of cognitive mechanisms in the way we process the information we access through the literature and collect during a clinical encounter. It has been argued that mindful awareness of type 1 and type 2 processes [15] may help us improve our decision-making processes. Our model provides the salient outline of these processes and how these processes can be effectively approached in clinical practice and education.

We demonstrated the applicability of our approach in a hypothetical case study in which a decision maker is considering radical prostatectomy for a patient who has elevated PSA and abnormal DRE. As the decision maker is uncertain whether a radical prostatectomy is the appropriate action for the particular patient, he also considers a prostate biopsy. Our results demonstrated the inability of EUT to model the preferences and attitudes of individual decision makers towards treatment and diagnostic testing. On the other hand, the DPM threshold provides a convincing explanation as to why treatment decisions vary between decision makers. We posit that this variation is contingent on the extent of activation of type 1 processes. Our main point here is that type 2 processes (as adhered to the EUT model) will always produce the same results, while it is type 1 processes that are unique to each decision-maker. In turn, it is the extent of activation of type 1 processes that can explain excessive ordering of diagnostic tests as well as overall variations in the treatment and diagnostic patterns documented in today’s clinical practice.

Our model has limitations. Even though it is a simple mathematical expression, its application is challenging since many of the model parameters are not easily elicited. Our suggestion, which was implemented in this paper, is to use data from published literature to valuate outcomes under type 2 processes and the Dual Visual Analogue scales developed in [21,22] to valuate outcomes under type 1 processes. In fact, we have shown that it is possible to elicit these values [32] although we have not yet done it in the context of the diagnostic setting. Our next step is to perform decision-making experiments initially in simulated environments, through hypothetical scenarios, and later in real clinical environments. In both cases, we will compare the decisions predicted by the DPM model to the actual decisions physicians make.

Throughout this article we avoided assigning a clear role to the decision maker. We believe that our methodology can be used by physicians and/or by patients. It is our position, however, that medical decision-making is shared between physicians and their patients. In such setting physicians recommend alternative treatment strategies with their associated harms and benefits and the patients eventually agree with the recommendation. We envision our methodology as a part of a computerized decision support system operated by the physician to elicit the patient’s preferences towards alternative forms of treatment.

## Conclusions

We have extended the recently derived DPM for medical decision making to include a diagnostic testing. Our model has the potential to explain the discrepancies found between optimal and actual actions. Because it captures the salient elements of medical decision-making via few parameters, our model has offered an important didactic value for medical education. Future research involves testing our model in a simulated environment with a wide variety of healthcare professionals.

## Supporting Information

S1 Appendix Detailed derivations of Dual Processing thresholds. (DOCX)
