# Supplementary material for: Dual Processing Model for Medical Decision-Making: An Extension to Diagnostic Testing
Source: PLoS One. 2015 Aug 5;10(8):e0134800. doi: 10.1371/journal.pone.0134800 (PMC4526559; doi:10.1371/journal.pone.0134800)
Supplement: S1 Appendix — (DOCX) [file pone.0134800.s001.docx]

**Dual processing model for medical decision-making: An extension to diagnostic testing**

**Appendix**

**Derivation of the testing thresholds**

Considering the decision tree in Fig. 2 we have:

| **Decision** | **Weight** | **Outcome** | **Utility** | **Valuation** | **Probability** |
| --- | --- | --- | --- | --- | --- |
| $Rx$ | $\gamma$ | $x_{1}$ | $U_{I,1}$ | $x_{1}^{m_{I}}=Rg\left( x_{1} \right)=0$ | $\frac{1}{2}$ |
|  | $\gamma$ | $x_{2}$ | $U_{I,2}$ | $x_{2}^{m_{I}}=Rg\left( x_{2} \right)=U_{I,4}-U_{I,2}$ | $\frac{1}{2}$ |
|  | $\left( 1-\gamma\right)$ | $x_{1}$ | $U_{II,1}$ | $x_{1}^{m_{II}}=U_{II,1}$ | $p$ |
|  | $\left( 1-\gamma\right)$ | $x_{2}$ | $U_{II,2}$ | $x_{2}^{m_{II}}{=U}_{II,2}$ | $1-p$ |

Note that under Type I processes, every outcome with non-zero probability is assigned equal weight. Therefore, in the two alternative outcomes case each probability is considered equal to 0.5 [[12](#_ENREF_12), [53](#_ENREF_53)]. The expected valuation of the decision to treat (Rx) is computed as the summation of the valuations of each outcome:

$$V\left( Rx \right)=\frac{\gamma}{2}\left( U_{I,2}-U_{I,4} \right)+\left( 1-\gamma\right)\left[ pU_{II,1}+\left( 1-p \right)U_{II,2} \right]$$

| **Decision** | **Weight** | **Outcome** | **Utility** | **Valuation** | **Probability** |
| --- | --- | --- | --- | --- | --- |
| $NoRx$ | $\gamma$ | $x_{3}$ | $U_{I,3}$ | $x_{3}^{m_{I}}=Rg\left( x_{3} \right)=U_{I,1}-U_{I,3}$ | $\frac{1}{2}$ |
|  | $\gamma$ | $x_{4}$ | $U_{I,4}$ | $x_{4}^{m_{I}}=Rg\left( x_{4} \right)=0$ | $\frac{1}{2}$ |
|  | $\left( 1-\gamma\right)$ | $x_{3}$ | $U_{II,3}$ | $x_{3}^{m_{II}}=U_{II,3}$ | $p$ |
|  | $\left( 1-\gamma\right)$ | $x_{4}$ | $U_{II,4}$ | $x_{4}^{m_{II}}{=U}_{II,4}$ | $1-p$ |

The expected valuation of the decision not to treat (NoRx) is computed as the summation of the valuations of each outcome:

$$V\left( NoRx \right)=\frac{\gamma}{2}\left( U_{I,3}-U_{I,1} \right)+\left( 1-\gamma\right)\left[ pU_{II,3}+\left( 1-p \right)U_{II,4} \right]$$

| **Decision** | **Weight** | **Outcome** | **Utility** | **Valuation** | **Probability** |
| --- | --- | --- | --- | --- | --- |
| $T$ | $\gamma$ | $x_{1T}$ | $U_{I,1}-H_{I,T}$ | $x_{1T}^{m_{I}}=Rg\left( x_{1T} \right)=-H_{I,T}$ | $\frac{1}{4}$ |
|  | $\gamma$ | $x_{2T}$ | $U_{I,2}-H_{I,T}$ | $x_{2T}^{m_{I}}=Rg\left( x_{2T} \right)=U_{I,2}-U_{I,4}-H_{I,T}$ | $\frac{1}{4}$ |
|  | $\gamma$ | $x_{3T}$ | $U_{I,3}-H_{I,T}$ | $x_{3T}^{m_{I}}=Rg\left( x_{3T} \right)=U_{I,3}-U_{I,1}-H_{I,T}$ | $\frac{1}{4}$ |
|  | $\gamma$ | $x_{4T}$ | $U_{I,4}-H_{I,T}$ | $x_{4T}^{m_{I}}=Rg\left( x_{4T} \right)=-H_{I,T}$ | $\frac{1}{4}$ |
|  | $\left( 1-\gamma\right)$ | $x_{1T}$ | $U_{II,1}-H_{II,T}$ | $x_{1T}^{m_{II}}=U_{II,1}-H_{II,T}$ | $pS$ |
|  | $\left( 1-\gamma\right)$ | $x_{2T}$ | $U_{II,2}-H_{II,T}$ | $x_{2T}^{m_{II}}=U_{II,2}-H_{II,T}$ | $(1-p)(1-S_{p})$ |
|  | $\left( 1-\gamma\right)$ | $x_{3T}$ | $U_{II,3}-H_{II,T}$ | $x_{3T}^{m_{II}}=U_{II,3}-H_{II,T}$ | $p(1-S)$ |
|  | $\left( 1-\gamma\right)$ | $x_{4T}$ | $U_{II,4}-H_{II,T}$ | $x_{4T}^{m_{II}}=U_{II,4}-H_{II,T}$ | $\left( 1-p \right)S_{p}$ |

Note that under Type I processes, every outcome with non-zero probability is assigned equal weight. Therefore, in the four alternative outcomes case each probability is considered equal to 0.25 [[12](#_ENREF_12), [53](#_ENREF_53)]. The expected valuation of the decision to test (T) is computed as the summation of the valuations for each outcome:

$$V\left( T \right)=\frac{\gamma}{4}(U_{I,2}-U_{I,4}+U_{I,3}-U_{I,1})+\left( 1-\gamma\right)\left[ pSU_{II,1}+\left( 1-p \right)\left( 1-S_{p} \right)U_{II,2}+p\left( 1-S \right)U_{II,3}+\left( 1-p \right)S_{p}U_{II,4} \right]-\left( \gamma H_{I,T}+\left( 1-\gamma\right)H_{II,T} \right)$$

**Thresholds**

We define the benefits and harms under type 1 and 2 as follows: $B_{I}=U_{I,1}-U_{I,3}$, $H_{I}=U_{I,4}-U_{I,2}$ and $B_{II}=U_{II,1}-U_{II,3}$, $H_{II}=U_{II,4}-U_{II,2}$. We assume that and $H_{I}, H_{II}>0$

**Testing threshold**

We set $V\left( NoRx \right)=V\left( T \right)$, which results in:

$$\frac{\gamma}{2}\left( U_{I,3}-U_{I,1} \right)+\left( 1-\gamma\right)\left[ pU_{II,3}+\left( 1-p \right)U_{II,4} \right]=\frac{\gamma}{4}\left( U_{I,2}-U_{I,4}+U_{I,3}-U_{I,1} \right)$$

$$+\left( 1-\gamma\right)\left[ pSU_{II,1}+\left( 1-p \right)\left( 1-S_{p} \right)U_{II,2}+p\left( 1-S \right)U_{II,3}+\left( 1-p \right)S_{p}U_{II,4} \right]-\left( \gamma H_{I,T}+\left( 1-\gamma\right)H_{II,T} \right)$$

$$\frac{\gamma}{4}\left( H_{I}-B_{I} \right)+\left( 1-\gamma\right)\left[ pU_{II,3}+\left( 1-p \right)U_{II,4} \right]+\left( \gamma H_{I,T}+\left( 1-\gamma\right)H_{II,T} \right)=\left( 1-\gamma\right)\left[ pSU_{II,1}+\left( 1-p \right)\left( 1-S_{p} \right)U_{II,2}+p\left( 1-S \right)U_{II,3}+\left( 1-p \right)S_{p}U_{II,4} \right]$$

$$\left( 1-\gamma\right)\left( 1-S_{p} \right)H_{II}+\left( 1-\gamma\right)H_{IIT}+\frac{\gamma}{4}\left( H_{I}-B_{I} \right)+\gamma H_{I,T}=\left( 1-\gamma\right)p\left[ SB_{II}+\left( 1-S_{p} \right)H_{II} \right]$$

$$\frac{\left( 1-S_{p} \right)H_{II}+H_{II,T}}{SB_{II}+\left( 1-S_{p} \right)H_{II}}+\frac{\gamma}{4\left( 1-\gamma\right)}\frac{\left( H_{I}-B_{I} \right)}{SB_{II}+\left( 1-S_{p} \right)H_{II}}+\frac{1}{\left( 1-\gamma\right)}\frac{\gamma H_{I,T}}{SB_{II}+\left( 1-S_{p} \right)H_{II}}=p$$

The formula for the threshold between withholding the treatment and testing becomes:

$$p_{tt}=\frac{1+\frac{1}{1-S_{p}}\frac{H_{II,T}}{H_{II}}}{1+\frac{S}{1-S_{p}}\frac{B_{II}}{H_{II}}}-\frac{\gamma}{4\left( 1-\gamma\right)}\frac{B_{I}-H_{I}}{SB_{II}+\left( 1-S_{p} \right)H_{II}}+\frac{1}{\left( 1-\gamma\right)}\frac{\gamma H_{I,T}}{SB_{II}+\left( 1-S_{p} \right)H_{II}}$$

$$p_{tt}=\frac{1+\frac{1}{1-S_{p}}\frac{H_{II,T}}{H_{II}}}{1+\frac{S}{1-S_{p}}\frac{B_{II}}{H_{II}}}+\frac{1}{4\left( 1-\gamma\right)\left( 1+\frac{S}{1-S_{p}}\frac{B_{II}}{H_{II}} \right)}\left( \gamma\frac{H_{I}-B_{I}}{\left( 1-S_{p} \right)H_{II}}+4\frac{\gamma H_{I,T}}{\left( 1-S_{p} \right)H_{II}} \right)$$

$$p_{tt}=\frac{1+\frac{1}{1-S_{p}}\frac{H_{II,T}}{H_{II}}}{1+\frac{S}{1-S_{p}}\frac{B_{II}}{H_{II}}}\left[ 1+\frac{\gamma}{4\left( 1-\gamma\right)\left( 1-S_{p} \right)\left( 1+\frac{1}{1-S_{p}}\frac{H_{II,T}}{H_{II}} \right)}\left( \frac{H_{I}}{H_{II}}\left( 1-\frac{B_{I}}{H_{I}} \right)+4\frac{H_{I,T}}{H_{II}} \right) \right]$$

$${p_{tt}=p}_{tt}(EUT)\left[ 1+\frac{\gamma}{4\left( 1-\gamma\right)\left( 1-S_{p} \right)\left( 1+\frac{1}{1-S_{p}}\frac{H_{II,T}}{H_{II}} \right)}\left( \frac{H_{I}}{H_{II}}\left( 1-\frac{B_{I}}{H_{I}} \right)+4\frac{H_{I,T}}{H_{II}} \right) \right]$$

*To constrain the values of* $p_{tt}\in[0,1]$*, we modify the* $p_{tt}$ *formula such as:*

$$p_{tt}=\min\left\{ p_{tt,EUT}\left[ 1+\frac{\gamma}{4\left( 1-\gamma\right)\left( 1-S_{p} \right)\left( 1+\frac{1}{1-S_{p}}\frac{H_{II,T}}{H_{II}} \right)}\left( \frac{H_{I}}{H_{II}}\left( 1-\frac{B_{I}}{H_{I}} \right)+4\frac{H_{I,T}}{H_{II}} \right) \right],1 \right\}, for \gamma\in[0,1)$$

*where* $p_{tt,EUT}\in[0,1]$ *is the EUT based testing threshold.*

**Treatment threshold**

We set $V\left( Rx \right)=V\left( T \right)$ which results in:

$$\frac{\gamma}{2}\left( U_{I,2}-U_{I,4} \right)+\left( 1-\gamma\right)\left[ pU_{II,1}+\left( 1-p \right)U_{II,2} \right]=\frac{\gamma}{4}\left( U_{I,2}-U_{I,4}+U_{I,3}-U_{I,1} \right)$$

$$+\left( 1-\gamma\right)\left[ pSU_{II,1}+\left( 1-p \right)\left( 1-S_{p} \right)U_{II,2}+p\left( 1-S \right)U_{II,3}+\left( 1-p \right)S_{p}U_{II,4} \right]-\left( \gamma H_{I,T}+\left( 1-\gamma\right)H_{II,T} \right)$$

$$\frac{\gamma}{4}\left( B_{I}-H_{I} \right)+\left( \gamma H_{I,T}+\left( 1-\gamma\right)H_{II,T} \right)=\left( 1-\gamma\right)\left[ -p\left( 1-S \right)B_{II}-\left( 1-p \right)S_{p}U_{II,2}+\left( 1-p \right)S_{p}U_{II,4} \right]$$

$$p\left[ \left( 1-S \right)B_{II}+S_{p}H_{II} \right]=S_{p}H_{II}-\frac{\gamma}{4\left( 1-\gamma\right)}\left( B_{I}-H_{I} \right)-\frac{\left( \gamma H_{IT}+\left( 1-\gamma\right)H_{II,T} \right)}{\left( 1-\gamma\right)}$$

$$p_{rx}=\frac{\frac{S_{p}H_{II}-H_{II,T}}{S_{p}H_{D}}}{\frac{\left( \left( 1-S \right)B_{II}+S_{p}H_{II} \right)}{S_{p}H_{II}}}+\frac{\gamma}{4\left( 1-\gamma\right)\left( \frac{\left( 1-S \right)B_{II}+S_{p}H_{II}}{S_{p}H_{II}} \right)}\left( \frac{\left( H_{I}-B_{I} \right)}{S_{p}H_{II}}-4\frac{H_{I,T}}{S_{p}H_{II}} \right)$$

$$p_{rx}=\frac{1-\frac{1}{S_{p}}\frac{H_{II,T}}{H_{II}}}{1+\frac{1-S}{S_{p}}\frac{B_{II}}{H_{II}}}\left[ 1+\frac{\gamma}{4\left( 1-\gamma\right)S_{p}\left( 1-\frac{1}{S_{p}}\frac{H_{II,T}}{H_{II}} \right)}\left( \frac{H_{I}}{H_{II}}\left( 1-\frac{B_{I}}{H_{I}} \right)-4\frac{H_{I,T}}{H_{II}} \right) \right]$$

$${p_{rx}=p}_{rx,EUT}\left[ 1+\frac{\gamma}{4\left( 1-\gamma\right)S_{p}\left( 1-\frac{1}{S_{p}}\frac{H_{II,T}}{H_{II}} \right)}\left( \frac{H_{I}}{H_{II}}\left( 1-\frac{B_{I}}{H_{I}} \right)-4\frac{H_{I,T}}{H_{II}} \right) \right]$$

*To constrain the values of* $p_{rx}\in[0,1]$*, we modify the* $p_{rx}$ *formula such as:*

$$p_{rx}=\min\left\{ p_{rx,EUT}\left[ 1+\frac{\gamma}{4\left( 1-\gamma\right)S_{p}\left( 1-\frac{1}{S_{p}}\frac{H_{II,T}}{H_{II}} \right)}\left( \frac{H_{I}}{H_{II}}\left( 1-\frac{B_{I}}{H_{I}} \right)-4\frac{H_{I,T}}{H_{II}} \right) \right],1 \right\}, for \gamma\in[0,1)$$

*where* $p_{rx,EUT}\in[0,1]$ *is the EUT based testing threshold.*

**Special Case:** $\gamma=1$

When $\gamma=1$, equations 1, 2, and 3 of the main manuscript are undefined. However, we can still identify the optimal decision by re-deriving the equations for $p_{t},p_{tt},$ and $p_{rx}$ using $\gamma=1$ in each strategy valuation function. The strategy with greatest valuation (see Fig 3) corresponds to the optimal decision. The valuations for each strategy are:

$V\left( NoRx \right)=\frac{1}{2}\left( U_{I,3}-U_{I,1} \right)=-\frac{1}{2}B_{I}$ (A1)

$V\left( Rx \right)=\frac{\gamma}{2}\left( U_{I,2}-U_{I,4} \right)+\left( 1-\gamma\right)\left[ pU_{II,1}+\left( 1-p \right)U_{II,2} \right]=-\frac{1}{2}H_{I}$ (A2)

$V\left( T \right)=\frac{1}{4}\left( U_{I,2}-U_{I,4}+U_{I,3}-U_{I,1} \right)-H_{I,T}=-\frac{1}{4}H_{I}-\frac{1}{4}B_{I}-H_{I,T}$ (A3)

1. For equation 1 of the main manuscript, which is used to decide between treating and not treating, we choose to treat when $(A2)\geq(A1) \to V\left( Rx \right)\geq V\left( NoRx \right)\to B_{I}\geq H_{I}$ otherwise we choose not to treat .
2. Similarly, for equation 2 of the main manuscript, which is used to decide between testing and no treatment, we choose no treatment when $(A1)\geq(A3)\to V\left( NoRx \right)\geq V\left( T \right)\to B_{I}\leq H_{I}+4H_{I,T}$. Conversely, we choose testing when $B_{I}>H_{I}+4H_{I,T}$.
3. Finally, for equation 3 of the main manuscript, which is used to decide between treating and testing, we choose treatment when $(A2)\geq(A3)\to V\left( Rx \right)\geq V\left( T \right)\to B_{I}\geq H_{I}-4H_{I,T}$ and we choose testing otherwise.

Since $H_{I}-4H_{I,T}\leq H_{I}+4H_{I,T}$, items 2 and 3 above can be combined as follows:

- From item 2, we choose testing when $B_{I}>H_{I}+4H_{I,T}$. However, if $B_{I}>H_{I}+4H_{I,T}$ then $B_{I}>H_{I}-4H_{I,T}$. Therefore, from item 3 above we can skip testing and choose treatment instead.
- From item 3, we choose testing when $B_{I}<H_{I}-4H_{I,T}$. However, if $B_{I}<H_{I}-4H_{I,T}$ then $B_{I}<H_{I}+4H_{I,T}$. Therefore, from item 2 above, we can skip testing and choose not to treat instead.

Note that whenever ${H_{I}-4H_{I,T}<B}_{I}<H_{I}+4H_{I,T}$, we are indifferent between picking treatment or no treatment, but since we don’t want to test, we can simply assume $H_{I,T}=0$ and base our decision on the comparison of $B_{I}$ and $H_{I}$.

To summarize, when $\gamma=1$, we choose treatment when $B_{I}\geq H_{I}-4H_{I,T}$ and choose no treatment if $B_{I}\leq H_{I}+4H_{I,T}$.
